# Management of traumatic cervical epidural hematoma in patients on Xa-inhibitors: a case report and review of the literature

**DOI:** 10.1186/s13256-023-04069-8

**Published:** 2023-11-07

**Authors:** Robert Dang, Leo Issagholian, Tegan Schmidt, Pasha Raoufi, Cameron C. Neeki, Michael M. Neeki

**Affiliations:** 1 Department of Emergency Medicine, Arrowhead Regional Medical Center, 400 N. Pepper Ave, Suite # 107, Colton, CA 92324 USA; 2 California University of Science and Medicine, Colton, CA USA; 3 Department of Surgery, Arrowhead Regional Medical Center, Colton, CA USA

**Keywords:** Trauma, Epidural hematoma, Anticoagulation reversal, Thromboelastogram, Factor Xa inhibitor, Prothrombin concentrate, Case report

## Abstract

**Background:**

Cervical epidural hematoma (CEH) is defined as a collection of blood in the suprameningeal space. Mechanisms of this rare pathology include spontaneous, postsurgical, and traumatic as the main subtypes. This unique case of traumatic CEH represents an even smaller subset of these cases. Management varies by symptom presentation, mechanism of injury, and other contraindications.

**Case presentation:**

This case presents a 32 year old African American female on an oral anticoagulant sustaining traumatic cervical hematoma after a motor vehicle collision. Patient complained of neck, abdominal, and back pain. Imaging revealed a cervical spinal hematoma at the level of C3–C6. This case discusses the management of CEH for the general population and in the setting of anticoagulation.

**Conclusion:**

Management of each case of CEH must be carefully considered and tailored based on their symptom presentation and progression of disease. As the use of anticoagulation including factor Xa inhibitors becomes more prevalent, there is greater need to understand the detailed pathophysiological aspect of the injuries. Targeted reversal agents such as Prothrombin Concentrate can be used for conservative treatment. Adjunct testing such as thromboelastogram can be used to help guide management.

## Background

Traumatic cervical epidural hematoma (CEH) is described as a collection of blood in the suprameningeal space of the outermost portion within the spinal canal [1]. It is hypothesized that damage to the posterior epidural venous plexus during a hyperextension or hyperflexion injury may contribute to the pathophysiology of CEH [2]. Similar complications have been described during whiplash injuries in motor vehicle collisions or cervical trauma resulted from a fall [1, 2].

CEH is rare with an annual incidence of 0.1 in 100,000 patients and presents in 0.63% of trauma patients annually [1]. Presence or development of CEH does not always correlate with the severity of the injuries and may present with a delay in the onset of symptoms, often being missed on initial imaging [2–7]. In addition, there is a higher prevalence (72–78%) in males at the later part of their life with a stronger association with traumatic events [6].

Complications of a CEH may include spinal cord compression and compromised neural tracts responsible for somatosensory and motor function. These symptoms may progress to lasting paresis or even death [2, 5]. Though the incidence of cervical spinal cord compression in the setting of CEH has been reported in as low as 2.4%, patients on anticoagulants involved in traumatic injuries are more likely to sustain extensive bleeding resulting in cord compression [6]. Symptoms mimicking Brown-Sequard Syndrome have also been noticed as an initial presentation of CEH with hemiparesis, ipsilateral loss of vibratory sensation, and contralateral loss of pain sensation [8]. Presenting symptoms may include cervical pain, stiffness, and ecchymosis, which make CEH difficult to detect [2, 6, 9].

Initial imaging studies may include computerized tomography (CT) scan or CT angiography which may reveal signs of epidural bleeding or early hematoma formation, however less than 25% of CEH cases are identified on these initial scans. In the setting of new, persistent, or worsening symptoms, CEH is often noted on the subsequent CT or magnetic resonance imaging (MRI) [2, 3, 5]. These studies may not be ordered in the setting of absence of neurological symptoms. Concerningly, this may allow occult advancement and growth of an epidural bleed until manifestation of neurologic symptoms, whereas earlier evaluation and intervention may have promoted a better prognosis.

This case presents a unique patient on a newer generation of oral factor Xa inhibitors involved in a motor vehicle collision sustaining CEH. Furthermore, this report explores the current literature in hope of elucidating an in-depth review of various approaches in the management of traumatic CEH.

## Case presentation

### Patient information

**Fig. 1:**
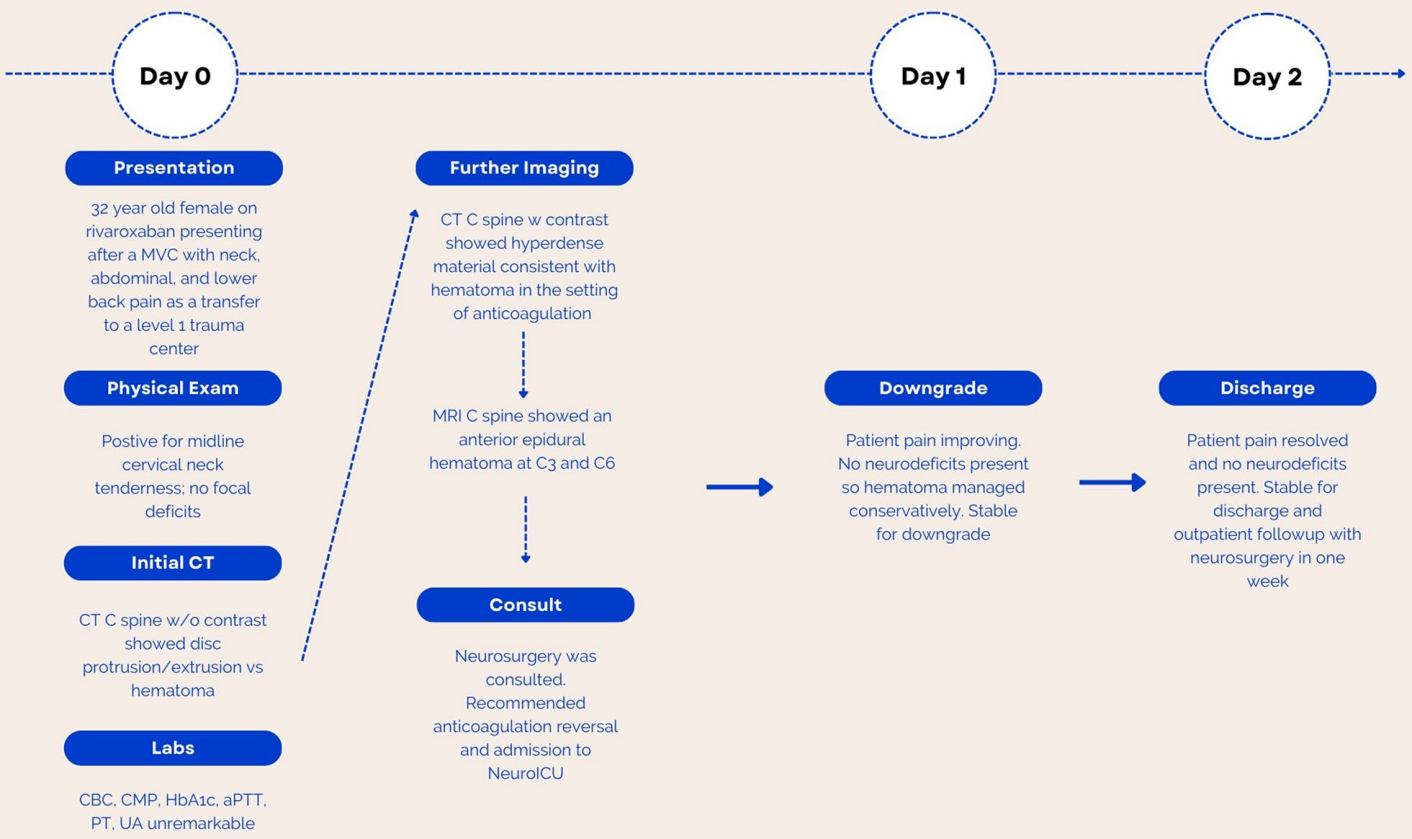
**Patient Hospital Timeline**: Timeline of patient's health care from admission to discharge

A 32-year-old African American female with a past medical history of lupus anticoagulant syndrome on oral Rivaroxaban (Xarelto) was transferred from a local hospital to a regional trauma center for a higher level of care after a motor vehicle collision. The patient was a restrained driver who struck a pole at approximately twenty miles per hour. She was able to self-extricate and ambulate on the scene but did not recall the details of the incident. She complained of neck, abdomen, and lower back pain to emergency medical services at the scene. During the initial evaluation at the local hospital, the patient underwent a series of imaging studies that included CT of the brain, cervical spine, chest, abdomen, and pelvis without contrast. Imaging studies revealed abnormal findings at the cervical spine C3–C4 and C4–C5 spinal cord levels consistent with disc protrusion/extrusion or possible hematoma. There were also traces of free fluid noted in the pelvic cavity. Initial findings prompted the transfer to the regional trauma center by aeromedical transportation for a higher level of care. Patient’s course is detailed in Fig. 1.

### Clinical findings

On arrival at the trauma center, she was somnolent and complained of nausea in addition to the neck and lower back pain. Her initial vitals included a blood pressure of 119/88 mm of mercury (mmHg), heart rate of 104 beats per minutes, temporal temperature of 36.6 °C (97.8 °F), respiratory rate of 22 breaths per minute and oxygen saturation of 99% on 2 L supplemental nasal cannula oxygen. Her past medical history was significant for lupus anticoagulant syndrome with prior history of deep vein thrombosis, anxiety, depression, attention deficit hyperactivity disorder, and insomnia. Her home medications included Rivaroxaban, Bupropion hydrochloride, Dextroamphetamine sulfate, Lamotrigine, and Quetiapine fumarate. Her social history was significant for smoking a pack of cigarettes every 3 days along with three to four glasses of wine weekly. She denied the use of any illicit drugs. On physical examination, the patient was alert, oriented, and calm with an initial Glasgow Coma Scale of 15. The patient was able to move all four extremities spontaneously. A cervical collar was placed at the initial hospital and was maintained during transportation. The patient had mild posterior cervical tenderness at the level of C3–C5. She had mild epigastric abdominal tenderness without guarding, rebound, or rigidity. The patient did not have any focal neurological deficits during initial or follow up exams. The rest of her physical exam was unremarkable.

### Diagnostic assessment

**Fig. 2:**
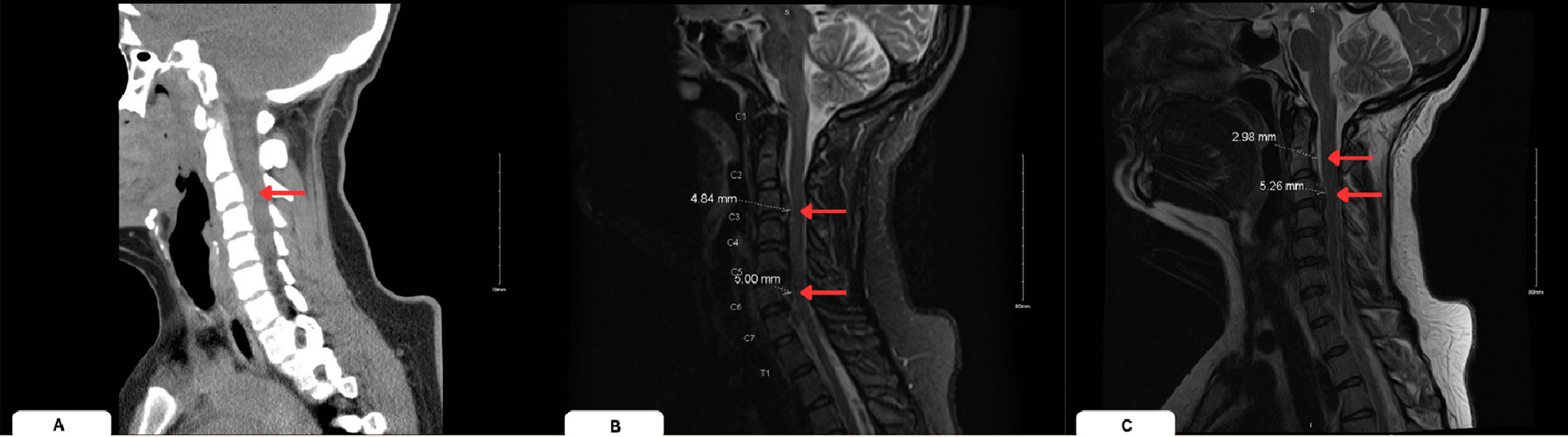
**A** Sagittal section of computerized tomography cervical spine without contrast displaying anterior cervical epidural hematoma at the level of C3 to C6 (left, red arrow), **B** Sagittal section of T2 weighted magnetic resonance imaging short tau inversion recovery displaying anterior cervical epidural hematoma at the level of C3 and C6 (middle, red arrows), **C** Sagittal section of T2 weighted magnetic resonance imaging American college of radiology displaying anterior cervical epidural hematoma

**Fig. 3:**
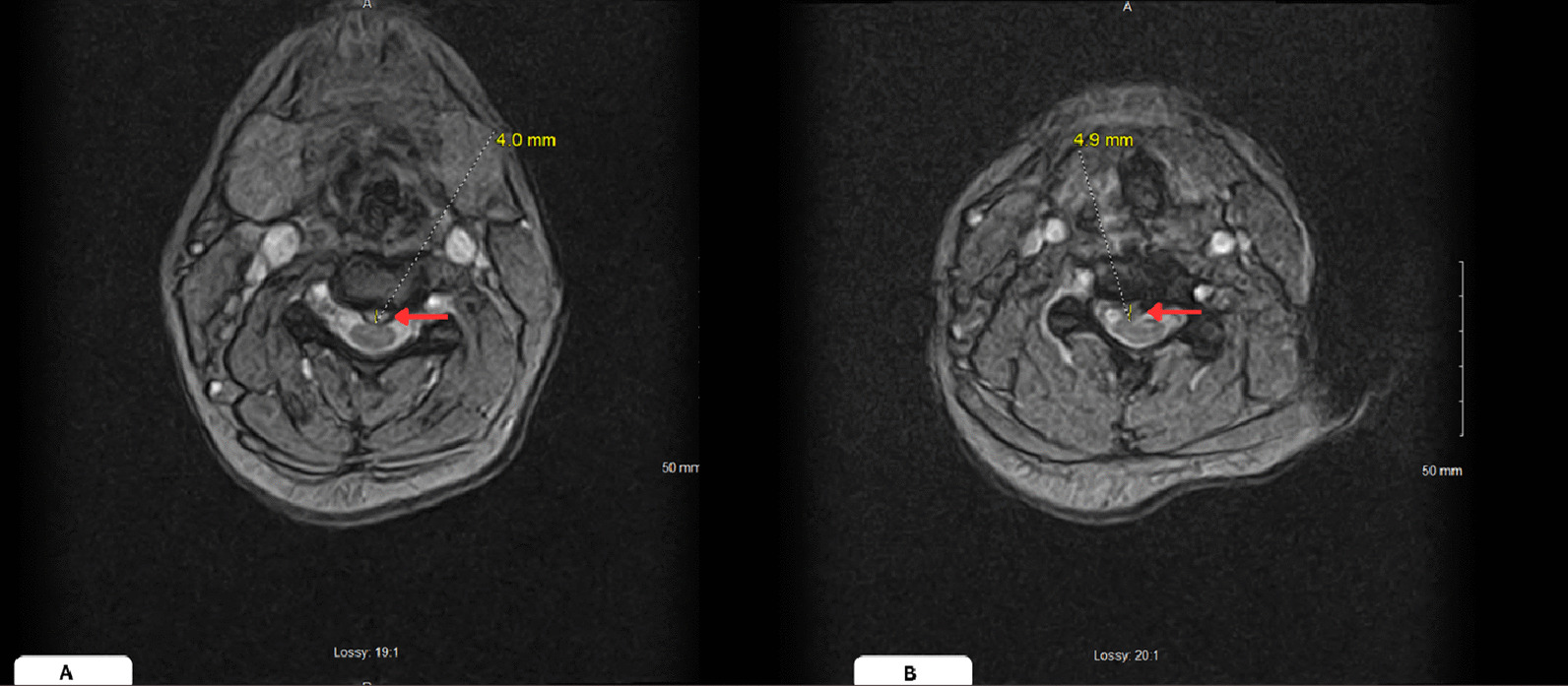
**A** Axial section of T2 weighted magnetic resonance imaging gradient echo displaying anterior cervical epidural hematoma at the level of C3 (left, red arrow). **B** Axial section of T2 weighted magnetic resonance imaging gradient echo displaying anterior cervical epidural hematoma at the level of C6 (right, red arrow)

The patient’s laboratory evaluations were grossly unremarkable on initial evaluation. These laboratory evaluations included complete blood count, basic metabolic panel, hemoglobin A1C, activated partial thromboplastin time, prothrombin time, and urinalysis. Urine toxicology was positive for marijuana and her serum ethanol level was 0.047 mg per deciliter. Repeat imaging studies at the trauma center included CT scans of the brain without contrast, and CT scan of cervical spine with intravenous (IV) contrast. The CT scan of the cervical region revealed a hyperdense material consistent with hematoma in the setting of anticoagulation (Fig. 2). Subsequently, the patient underwent MRI of the cervical spine without contrast that indicated an anterior epidural soft tissue thickening at C2–C3 region consistent with an epidural hematoma (Fig. 3).

### Therapeutic intervention/outcomes

The neurosurgical service was consulted, and the patient was evaluated by their team. The consultant recommended anticoagulation reversal with administration of IV prothrombin complex concentrate (Kcentra) and cervical precautions with continued cervical collar placement. The patient was admitted to the surgical intensive care unit with hourly neurological evaluations, head of bed at > 30°, and systolic blood pressure maintained between 100 and 140 mmHg at all times. The standard hospital anticoagulants were avoided in this case and sequential compression devices were applied for deep vein thrombosis prophylaxis. The patient was downgraded within 24 hours of admission and eventually discharged after 48 hours of hospitalization. There were no adverse or unanticipated events. The patient was instructed to follow up with a spine specialist in 1 week, to avoid anticoagulation for 1 month, and to maintain the cervical collar in place until reevaluation by the specialist. The patient then followed up uneventfully with their primary care at an outside facility not within the hospital network. It is unclear when or if the patient resumed their anticoagulation regimen.

## Discussion

This case highlights successful nonoperative treatment in the setting of traumatic CEH. Patient’s primary risk factors included history of factor X inhibitor anticoagulation and trauma. The literature review suggested that there are independent risk factors for the development of CEH [5]. One recent phenomenon is the prevalence of using anticoagulants among the aging population for conditions such as atrial fibrillation, cerebrovascular diseases, and coronary artery disease [5]. Disruption of coagulative properties, resulting from iatrogenic and paraneoplastic factors, also constitute increased risks of the development of hematoma or bleeding. Absence of anticoagulation in the setting of a spinal epidural hematoma has been noted to be protective, with a 4–5 odds ratio of complete recovery whether surgically or non-operatively treated [10]. MRI has been recommended as the primary imaging of choice for diagnosis of the CEH, however, in this case, the initial signs of CEH were noted on cervical CT scan without IV contrast which is the most prevalent imaging study available at hospitals [3, 5].

Ricart and colleagues reported that up to 40% of CEHs occur in the postoperative setting or are sustained spontaneously [6]. In the event of trauma, one suggested etiology for CEH is distorted arteriovenous malformations within the epidural venous plexus [11]. While the exact mechanism is unclear for this case, the most readily apparent risk factor for CEH is the use of anticoagulants with synergistic effects of the traumatic event. Anticoagulation, liver failure, malignancy, pregnancy, and other coagulopathic states have been identified as risk factors for development of epidural bleeding [11]. Typical evaluation of coagulation status in a patient is by prothrombin time, activated partial thromboplastin time, and international normalized ratio (INR) [12, 13]. Elevated INR has been implicated in some studies as an important metric in the evaluation of traumatic CEH, though this patient in the current case did not suffer from a grossly elevated INR throughout the course of hospitalization [6]. Though elevated INR (by a median increase of approximately 0.5) has been reported in the use of Apixaban, however this may not place patients in a typical therapeutic range [12, 13].

Rivaroxaban is a factor Xa inhibitor and this specific class of novel oral anticoagulants has been found as a risk factor for traumatic CEH [5]. In the setting of acute bleeding events such as CEH, reversal agents may be administered as a stabilizing measure. The selection of reversal agents may be specific or nonspecific, ranging from platelet or fresh frozen plasma transfusions to prothrombin concentrate for Rivaroxaban reversal [14]. Other potential therapies may include platelet promoting factors like Desmopressin in the treatment of CEH in the setting of Aspirin or Clopidogrel use by the patients [15]. An essential step in the management of these cases is to address the underlying coagulopathy, avoid complications, and prevent worsening of the patient’s conditions. An anticoagulant holiday was recommended to this patient, which is consistent with previous case reports [8]. Consideration of the past medical history necessitating patients on anticoagulation raises the question of appropriate timeline of anticoagulant cessation to resumption. The patient in this case had a history of lupus anticoagulant syndrome, however other cases of reported CEH in the literature included underlying histories such as atrial fibrillation or malignancies [4]. Review of literature indicated that in patients with lupus anticoagulant syndrome, cessation of anticoagulation between 9 and 23 months was associated with venous thromboembolism in 15–40% of patients [16]. With this consideration in mind, the 1 month recommendation for this patient was likely safe and beneficial with no further hemorrhage or complication that was reported at the follow up visit. There is no clear standard recommendation per the existing literature regarding the timeline for anticoagulant cessation in the setting of spinal epidural hematomas. However, close follow up with a specialist in such a complex pathology is likely beneficial.

The current standard for treatment of symptomatic CEH with neurological symptom manifestation is decompression surgery by hemi or full decompressive laminectomy [17]. An alternative operative approach suggested in the literature is laparoscopic hematoma evacuation [18]. Previous published case reports have identified nonsurgical treatment as an option in the absence of cord compression symptoms, such as unilateral or bilateral extremity weakness, loss of bowel or bladder control, or sensory deficit [5, 19]. A published case series by Duffill and colleagues, reported 4 cases of spontaneous CEH that were treated nonoperatively despite some neurological manifestations such as weakness and paresthesias [20]. Rationale by the authors for electing to treat nonoperatively included rapid improvement of symptoms over a period of several hours after onset as well as poor surgical candidate [20].

Musha and colleagues noted that even in patients who develop symptoms of paralysis in the setting of acute spinal epidural hematoma, improvement of symptoms within 11 hours may serve as a moderately effective indicator of complete nonoperative recovery [10]. Patients treated operatively before 12 hours of onset of symptoms had good prognosis, however patients with delayed treatment up to 24 hours still saw improvement [21]. In this case, no neurological deficit was observed when the CEH was found on imaging, and as a result, a conservative treatment route with prothrombin concentrate (Kcentra) was utilized.

Thromboelastogram (TEG) is an effective tool available to analyze the status of a patient’s state of coagulopathy and is employed in the major emergency or trauma centers [22]. Reported values include fibrin formation time and clot formation time. TEG has been validated as a practical tool to monitor for targeted anticoagulation such as in the setting of factor II and factor X inhibitor use [23]. The use of TEG is not as widespread as conventional markers like Prothrombin Time, activated Partial Thromboplastin Time, or INR, however there has been several studies detailing its use and benefits at trauma centers [24] Additionally, TEG has been utilized in monitoring patients on multiple forms of anticoagulation while undergoing targeted therapy using Factor II reversal with Idarucizumab (Praxbind) [25]. A similar case has been reported in reversal of a Factor X inhibitor, Apixaban, as detected on TEG [25]. Decision to reverse anticoagulation guided by TEG can be both cost effective in avoiding unnecessary concentrated factor transfusion or the reversal agents in otherwise therapeutic anticoagulation for conditions like atrial fibrillation or thromboembolic events [26]. Utilization of this technology has proven effective in acute care of trauma patients with a bleeding event.

## Conclusion

Monitoring the severity and progression of CEH is crucial to determine the appropriate treatment. As the use of anticoagulation including factor Xa inhibitors becomes more prevalent, there is greater need to understand the detailed pathophysiological aspect of the injuries, adjunct testing and treatment options to improve the quality of the patient care.

## Data Availability

The datasets used and/or analyzed during the current study are available from the corresponding author on reasonable request.

## Abbreviations

CEH: Traumatic cervical epidural hematoma
CT: Computerized tomography
mmHg: Millimeters of mercury
INR: International normalized ratio
TEG: Thromboelastogram
IV: Intravenous
